# TriCurin, a novel formulation of curcumin, epicatechin gallate, and resveratrol, inhibits the tumorigenicity of human papillomavirus-positive head and neck squamous cell carcinoma

**DOI:** 10.18632/oncotarget.10620

**Published:** 2016-07-16

**Authors:** Longzhu Piao, Sumit Mukherjee, Qing Chang, Xiujie Xie, Hong Li, Mario R. Castellanos, Probal Banerjee, Hassan Iqbal, Ryan Ivancic, Xueqian Wang, Theodoros N. Teknos, Quintin Pan

**Affiliations:** ^1^ Department of Otolaryngology-Head and Neck Surgery, The Ohio State University Medical Center, Columbus, Ohio, USA; ^2^ Arthur G. James Cancer Hospital and Richard J. Solove Research Institute, The Ohio State University Comprehensive Cancer Center, Columbus, Ohio, USA; ^3^ Ph.D. Program in Biochemistry at the Graduate Center of the City University of New York, New York, USA; ^4^ Department of Chemistry and Center for Developmental Neuroscience, College of Staten Island, Staten Island, New York, USA; ^5^ Department of Pathology and Laboratory Medicine, Staten Island University Hospital, Northwell Health, Staten Island, New York, USA; ^6^ Division of Pharmaceutics, College of Pharmacy, The Ohio State University, Columbus, Ohio, USA; ^7^ Division of Research, Department of Medicine, Staten Island University Hospital, Northwell Health, Staten Island, New York, USA

**Keywords:** human papillomavirus, p53, head and neck cancer, curcumin, catechin

## Abstract

Head and neck squamous cell carcinoma (HNSCC) is the sixth most prevalent cancer worldwide with about 600,000 new cases diagnosed in the last year. The incidence of human papillomavirus-positive head and neck squamous cell carcinoma (HPV-positive HNSCC) has rapidly increased over the past 30 years prompting the suggestion that an epidemic may be on the horizon. Therefore, there is a clinical need to develop alternate therapeutic strategies to manage the growing number of HPV-positive HNSCC patients. TriCurin is a composition of three food-derived polyphenols in unique stoichiometric proportions consisting of curcumin from the spice turmeric, resveratrol from red grapes, and epicatechin gallate from green tea. Cell viability, clonogenic survival, and tumorsphere formation were inhibited and significant apoptosis was induced by TriCurin in UMSCC47 and UPCI:SCC090 HPV-positive HNSCC cells. Moreover, TriCurin decreased HPV16E6 and HPV16E7 and increased p53 levels. In a pre-clinical animal model of HPV-positive HNSCC, intra-tumoral injection of TriCurin significantly inhibited tumor growth by 85.5% compared to vehicle group (*P* < 0.05, *n* = 7). Our results demonstrate that TriCurin is a potent anti-tumor agent for HPV-positive HNSCC. Further development of TriCurin as a novel anti-cancer therapeutic to manage the HPV-positive HNSCC population is warranted.

## INTRODUCTION

Approximately 600,000 new cases of head and neck squamous cell carcinoma (HNSCC) are diagnosed worldwide each year [1, 2]. These cancers are strongly associated with certain environmental and lifestyle risk factors, such as tobacco and alcohol consumption. More recently, high-risk human papillomavirus (HPV) infection, in particular HPV16, has emerged as an etiologic factor for the development of HNSCC [3]. Overall, about 25% of all HNSCC are positive for HPV DNA. HPV-positive HNSCC displays clinical and molecular features that distinguish it from the traditional alcohol- and smoking-related HPV-negative HNSCC [4]. Retrospective and prospective studies have shown that HPV-positive HNSCC patients have better response rates and clinical outcomes than HPV-negative HNSCC patients [5]. Standard of care treatment paradigms consisting of chemotherapy and/or radiation for HNSCC are fairly toxic and associated with high morbidity and quality of life issues. Based on these concerns, there were discussions and now clinical trials designed to assess the impact of treatment de-intensification strategies for the HPV-positive HNSCC population. Alternatively, novel less-toxic therapeutic approaches need to be developed to add to the armamentarium to manage HPV-positive HNSCC patients.

Several pre-clinical studies reported that natural phenolic compounds have anti-HPV and anti-cancer activity [6, 7]. Plant phenols play an important role in cancer prevention. Epidemiologic studies show diets high in these polyphenol-rich foods, such as vegetables and fruits, are associated with reduced cancer rates [8]. Curcumin is a polyphenol extracted from the food spice turmeric. In China and India, turmeric has been used as medicinal herb to treat various ailments over the centuries. Numerous scientific reports have shown that curcumin has anti-cancer activity and targets numerous oncogenic pathways [9–12]. Our group demonstrated that curcumin eliminates various HPV-positive cervical carcinoma cell lines and is non-toxic to healthy tissue [13]. Resveratrol is a polyphenol found in grapes, it is also a potent antioxidant [14–17] with anti-inflammatory and cancer-preventive [18–21] properties. Studies reported that both curcumin and resveratrol displayed synergistic effects with cancer therapeutics [22–24]. Epicatechin gallate is one of the major catechins in green tea and also has shown anti-cancer activity on a variety of cell types [25]. Although pre-clinical studies show these polyphenols as promising anti-cancer compounds, clinical trials evaluating curcumin, epicatechins, and resveratrol have had only marginal results due to poor bioavailability and low target tissue uptake [26, 27].

To overcome the clinical challenge associated with developing polyphenols as a therapeutic, TriCurin, a triple polyphenol combination consisting of curcumin, epicatechin gallate, and resveratrol in specific stoichiometric proportions, was formulated to maximize anti-tumor activity. In this study, TriCurin was highly active to eliminate HPV-positive HNSCC cells *in vitro* and *in vivo*. Our work provides initial evidence that TriCurin may be a promising therapeutic to manage HPV-positive HNSCC patients.

## RESULTS

### TriCurin promotes a global anti-tumor response in HPV-positive HNSCC

**Figure 1:**
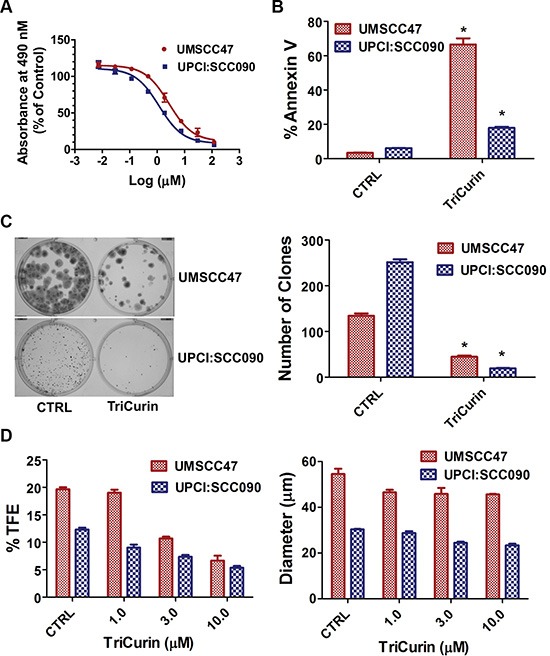
TriCurin promotes a global anti-tumor response in HPV-positive HNSCC (**A**) IC50. The MTS reagent was used to measure cell proliferation. Dose response curve and IC50 of TriCurin were generated using GraphPad Prism software. (**B**) Apoptosis. UMSCC47 and UPCI:SCC090 cells were treated with control or TriCurin (3 μM+). Cells were stained with FITC-Annexin V and propidium iodide and analyzed by flow cytometry. Data are presented as mean ± SEM. *P < 0.01, *n* = 3. (**C**) Clonogenic survival. UMSCC47 and UPCI:SCC090 cells were treated with control or TriCurin (3 μM+). The number of surviving clones was assessed by counting the number of clones under a microscope after crystal violet staining. A representative well for each experimental condition is shown and data are presented as mean ± SEM. *P < 0.01, *n* = 3. (**D**) Tumorsphere formation efficiency and diameter. UMSCC47 and UPCI:SCC090 cells were treated with control or TriCurin (1, 3, or 10 μM+) and cultured in ultralow attachment plates. Tumorsphere formation efficiency was calculated as the number of tumorspheres formed divided by the original number of cells seeded. Tumorsphere diameter was measured using NIS-Elements software. Data are presented as mean ± SEM. *P < 0.01, *n* = 3 for tumorsphere formation efficiency. *P < 0.05, *n* = 3 for tumorsphere diameter.

Two HPV16-positive HNSCC cell lines, UMSCC47 and UPCI:SCC090, were tested for growth inhibition using a dose range of TriCurin (Figure 1A). The IC50 was calculated to be 3.22 μM+ and 1.86 μM+ for UMSCC47 and UPCI:SCC090, respectively. TriCurin (3 μM+ for 72 hours) promoted apoptosis in both HPV16-positive HNSCC cell lines (Figure 1B). In comparison with vehicle, TriCurin induced 66.1 ± 3.6% apoptosis in UMSCC47 (*P* < 0.01, *n* = 3), and 11.8 ± 0.7% apoptosis in UPCI:SCC090 cells (*P* < 0.01, *n* = 3). Clonogenic survival assay was used to assess the clonal proliferation of surviving cells over a prolonged time-period. As shown in Figure 1C, clonogenic survival of UMSCC47 and UPCI:SCC090 cells was compromised with TriCurin treatment (3 μM+ for 24 hours); 66.7 ± 3.0% (*P* < 0.01, *n* = 3) inhibition for UMSCC47 and 92.4 ± 1.0% inhibition for UPCI:SCC090 (*P* < 0.01, *n* = 3). Moreover, the combination of TriCurin and radiation was more active (*P* < 0.01, *n* = 3) than either single intervention to ablate UMSCC47 cells (Supplementary Figure 1). Radiation (3 Gy) reduced clonogenic survival of UMSCC47 cells by 44.8 ± 1.7% whereas the TriCurin and radiation combination decreased the number of survival clones by 79.9 ± 3.6%. The ability of cells to form spheres under non-adherent culture conditions has been widely used as an *in vitro* assay to assess normal stem cells and cancer initiating cells (CICs). Our group showed that tumorspheres are enriched for CICs and a single tumorsphere has the potential to generate a bulky tumor *in vivo* [28]. Tumorsphere formation efficiency was reduced in a dose-dependent manner with TriCurin treatment (Figure 1D). At the highest concentration (10 μM+), TriCurin decreased tumorsphere formation efficiency by 66.1 ± 7.8% (*P* < 0.01, *n* = 3) in UMSCC47 and 56.7 ± 4.7% (*P* < 0.01, *n* = 3) in UPCI:SCC090. In addition, TriCurin reduced tumorsphere diameter by 16.0 ± 0.8% (*P* < 0.05, *n* = 3) in UMSCC47 and 19.4 ± 0.3% (*P* < 0.05, *n* = 3) in UPCI:SCC090 (Figure 1E).

### TriCurin reduces HPV16E6/E7 and enhances p53/Rb levels in HPV-positive HNSCC

**Figure 2:**
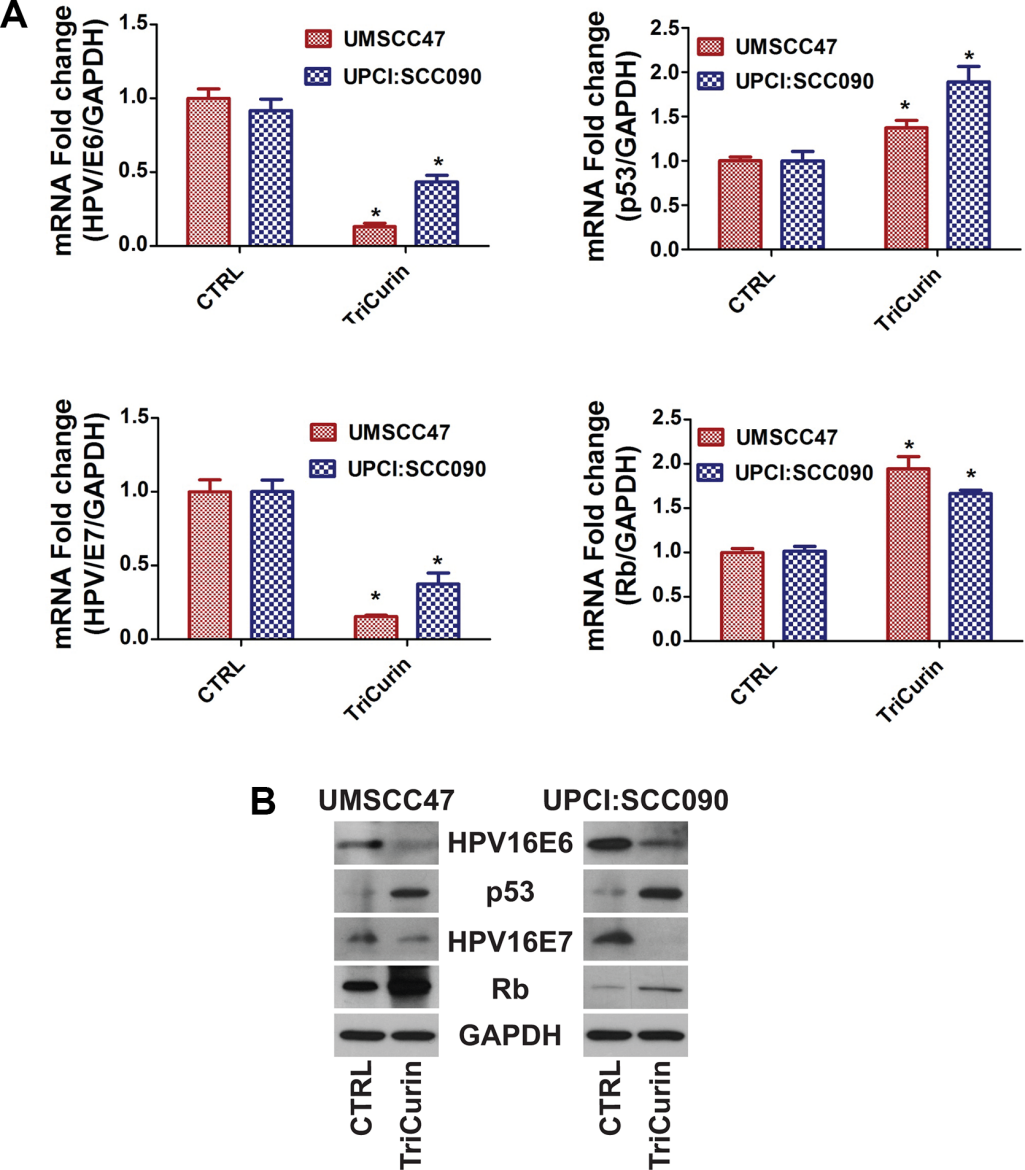
TriCurin reduces HPV16E6/E7 and enhances p53/Rb levels in HPV-positive HNSCC UMSCC47 and UPCI:SCC090 cells were treated with control or TriCurin (3 μM+ for 72 hours. (**A**) HPV16E6/E7 and p53/Rb mRNA expression. mRNA expression was determined by qRT-PCR. Data were normalized to GAPDH and presented as mean ± SEM. *P < 0.001, *n* = 5. (**B**) HPV16E6/E7 and p53/Rb protein levels. Protein levels were determined by immunoblot analyses.

It is well recognized that HPV16 oncogenes, E6 and E7, inactivates p53 and Rb, respectively, to drive HPV- induced carcinogenesis. shRNA-mediated knockdown of HPV16E6/E7 is sufficient to promote apoptosis and ablate HPV16-positive HNSCC cells [29]. Therefore, we determined if the global anti-tumor effect of TriCurin is mediated through suppression of HPV16E6/E7. In Figure 2, TriCurin treatment dramatically reduced HPV16E6 and HPV16E7 mRNA expression and protein levels in UMSCC47 and UPCI:SCC090 cells. Moreover, an increase in p53 and Rb levels were observed following TriCurin treatment. These results confirm that TriCurin targets HPV16E6/E7 resulting in an accumulation of p53 and Rb in HPV-positive UMSCC47 and UPCI:SCC090 cells.

### TriCurin retards tumor growth *in vivo*

**Figure 3:**
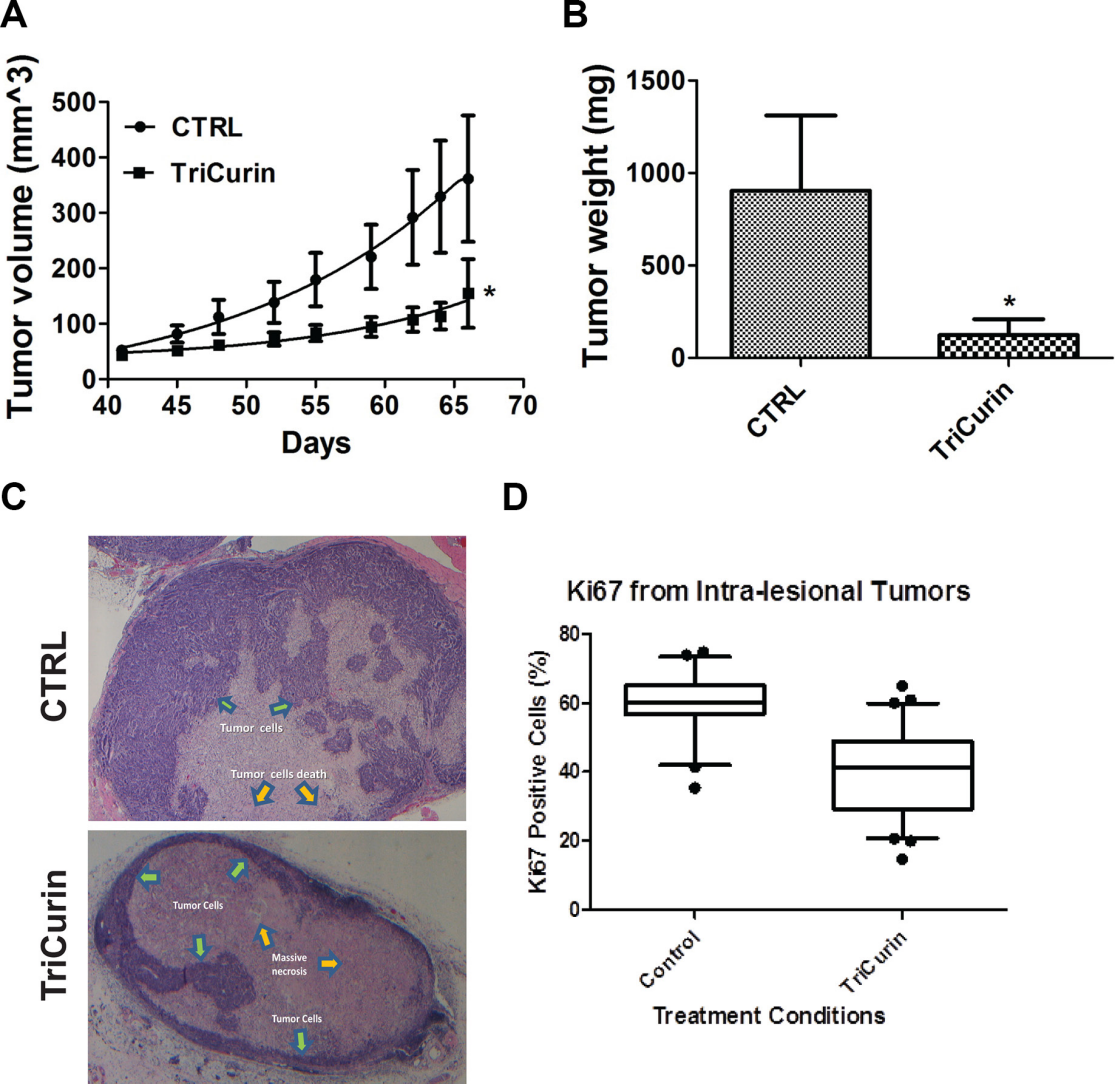
TriCurin retards tumor growth in a pre-clinical model of HPV-positive HNSCC (**A**) Tumor volume. UMSCC47 cells were implanted into the flanks of athymic nude mice and tumors were allowed to develop without treatment. At 41 days post-tumor cell implantation, mice with palpable tumors were randomly assigned to two treatment arms: vehicle or TriCurin (3×/week for 5 weeks). Tumors were measured using a digital caliper and tumor volumes were calculated. Data are presented as mean ± SEM. *P < 0.05, *n* = 7. (**B**) Tumor weight. Mice were sacrificed at the end of the treatment protocol and tumors were resected and weighed. Data are presented as mean ± SEM. *P < 0.01, *n* = 7. (**C**) H&E histopathology. A representative image from a tumor in the control or TriCurin-treated group is shown. Tumors from TriCurin-treated mice showed large necrotic areas (yellow arrows) with residual tumor cells (green arrows) in the periphery. Tumors from control-treated mice were multi-lobulated with large areas populated with residual tumor cells. (**D**) Ki67 staining. Typical areas for each case were photographed with a 20× objective lens and shown. The Ki67 labeling index was scored as the percentage of tumor cell nuclei showing definite nuclear immunoreactivity above the background level using Image J. Data are presented as mean ± SEM. *P < 0.003.

**Figure 4:**
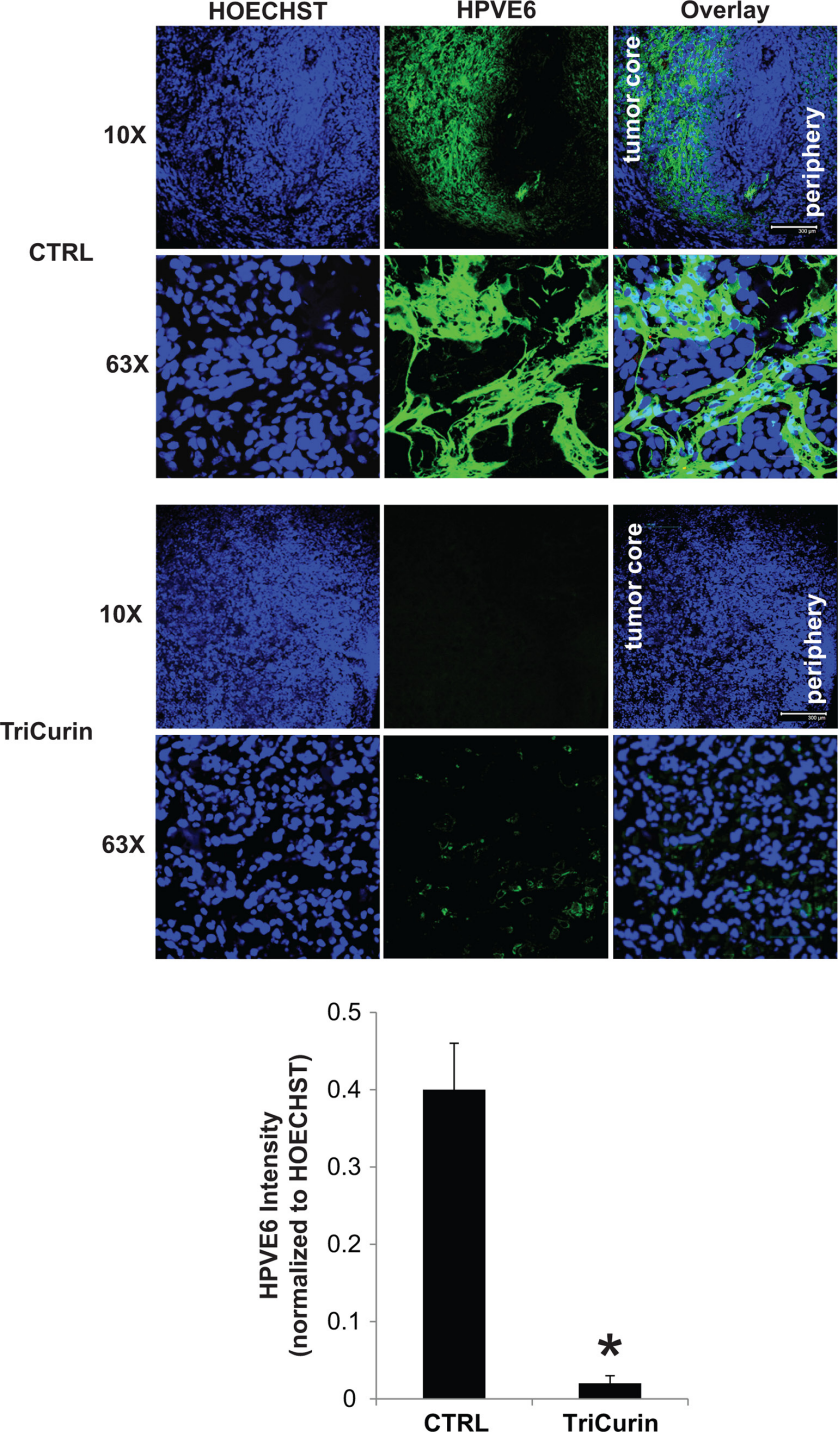
TriCurin reduces intra-tumoral levels of HPV16E6 Representative immunofluorescence images from a tumor in the control or TriCurin-treated group are shown (scale bar: 300 μm). Nuclear staining with HOECHST33342 was used to normalize for HPV16E6 staining intensity. Data are presented as mean ± SEM. *P < 0.001.

The *in vivo* activity of TriCurin was assessed in a pre-clinical mouse model of HPV16-positive HNSCC. UMSCC47 cells were implanted into the flanks of athymic nude mice and allowed to establish without treatment. At 41 days post-tumor cell implantation, mice with established tumors were randomly assigned to two treatment arms; vehicle or TriCurin (3×/week for 5 weeks). Intra-tumor therapy for the treatment of HNSCC has been previously proposed using gene therapy [30]. Though only limited effects have been seen in these phase 1 and 2 clinical trials [31], the feasibility of doing intra-tumor treatment was validated in the clinical setting. Intra-tumoral delivery of TriCurin inhibited tumor growth by 85.5% (*P* < 0.05, *n* = 7) (Figure 3A). At the end of the treatment protocol, mice were sacrificed and tumors were resected and weighed. TriCurin reduced the tumor weight by 86.3% (*P* < 0.01, *n* = 7); the mean tumor weight was 904.3 ± 203 and 123.5 ± 42.8 mgs for the vehicle and TriCurin, respectively (Figure 3B). Analysis of the H&E stained tumor sections from the TriCurin group showed that the majority of the tumor consisted of large necrotic areas with small regions of tumor cells present in the tumor periphery (Figure 3C). In contrast, as shown in Figure 3D, the tumors from the vehicle group were multi-lobulated and predominantly populated with tumor cells. The vehicle-treated tumors also had regions of necrosis but comparatively much less necrotic tissue than the TriCurin-treated tumors. Ki67 immunohistochemical staining showed that TriCurin treatment reduced the number of actively proliferating tumor cells by 19.9% (*P* < 0.003) compared to vehicle (Figure 3E). Furthermore, TriCurin dramatically reduced the intra-tumoral levels of HPV16E6 (94.9% inhibition, *P* < 0.001) (Figure 4).

## DISCUSSION

HNSCC encompasses many site-specific cancers, including oral cavity and oropharyngeal cancers. Alcohol and tobacco are the traditional risk factors for HNSCC and incidence rates are found to be higher in regions with high rates of alcohol and tobacco consumption [32]. During the past few decades, several countries have witnessed a decline in oral cavity cancer incidence due to a decline in tobacco use. However, Canada, Denmark, the Netherlands, Norway, Sweden, the United States, and the United Kingdom, have seen an increasing rate of oropharyngeal and oral cavity cancers despite declines in smoking rates since the 1980s. The increase in HNSCC incidence rate in these developed countries is attributed to HPV, in particular high-risk HPV16 [33]. It is estimated that by 2025, the number of new HPV-positive HSNCC cases will be similar if not greater than the number of new HPV-positive cervical cases. Thus, there is a critical need to develop novel therapeutic strategies to better manage the growing number of HPV-positive HNSCC population.

Polyphenols are present in foods and beverages of plant origin (fruits, vegetables, cereals, herbs, spices, legumes, nuts, olives, chocolate, tea, coffee, and wine) and are the most abundant antioxidants in the human diet [34]. Polyphenols show many beneficial effects on human health including antimicrobial, anti-inflammatory, anti-viral, anti-tumoral, and immunomodulatory activities [35–39]. One of the most potent and promising anti-tumor polyphenols reported in preclinical studies is curcumin [10, 11]. Curcumin has been shown to promote apoptosis through PARP-dependent and PARP-independent pathways. Recent work revealed a role for apoptosis inducing factor in curcumin-mediated apoptosis [40]. These results demonstrate that curcumin-mediated cell death is complex and may involve multiple apoptotic pathways. Our group demonstrated that curcumin effectively eliminates various HPV-positive cervical carcinoma cell lines *in vitro*; prior to cell death, curcumin simultaneously decreased HPVE6 levels and induced the expression of p53 [13]. The anti-tumor effect of curcumin was not HPV-type specific, showing activity against high-risk HPV genotypes 16, 18, and 68. Similarly, the polyphenols extracted from green tea, catechins, have shown potent activity against HPV-positive carcinoma cells. Catechins inhibit cell proliferation in a dose-dependent manner and directly inhibit HPVE6 and HPVE7 [41]. Resveratrol is another polyphenol with anti-tumor activity and promotes cell cycle arrest and apoptosis in HPV-positive cervical carcinoma cells [7]. These plant-derived polyphenols demonstrated promising pre-clinical efficacy; however, curcumin, catechins, and resveratrol have not advanced beyond clinical trials due to limitations, such as rapid metabolism and low target tissue bioavailability.

Our previous work demonstrated that single-agent curcumin, epicatechin gallate, or resveratrol inhibits proliferation and E6 levels in HPV-positive HeLa cervical carcinoma [42, 43]. Combination therapy of curcumin, epicathechin gallate, and resveratrol at their IC50 doses (16 μ*M*, 16 μ*M*, and 65 μ*M*, respectively) showed general toxicity and did not discriminate between HeLa cells and normal fibroblasts [42]. Comprehensive single-, double-, and triple-agent studies revealed that curcumin, epicatechin gallate, and resveratrol at a defined molar ratio of 4:1:12.5 (TriCurin) was selective to ablate HeLa cells and furthermore, more active than each individual component or the doublet of curcumin and epicatechin gallate or curcumin and resveratrol [42]. Moreover, intra-lesional administration of TriCurin was highly active and inhibited *in vivo* tumor growth of HPV16E6/E7, c-Ha-ras-expressing TC-1 cells by 80% [42]. In the present study, TriCurin exhibited high anti-tumor activity *in vitro* and *in vivo* against HPV16-positive HNSCC cells. HPV16E6 and HPV16E7 levels were dramatically reduced, whereas, p53 and Rb levels were increased with TriCurin treatment. Since HPV16E6 and HPV16E7 oncogenic proteins are essential for cell transformation and tumor cell maintenance, we suggest that a potential mechanism of action of TriCurin in HPV16-positive HNSCC cells is through reduction of the HPV16E6/E7 viral proteins. Our *in vivo* data show that intra-tumor delivery of TriCurin results in a large amount of tumor cell death in the region of the injection site. Furthermore, tumor cells in the periphery of the tumor showed an altered phenotype with decrease Ki67 staining and a significant decrease in the levels of the viral oncogenic HPV16E6 protein. Radiation is a standard of care for HNSCC patients and the observation that TriCurin potentiates the anti-tumor efficacy of radiation provides initial evidence that the TriCurin and radiation combination should be extensively explored in future studies. Taken together, our findings provide initial evidence that TriCurin may be an alternative therapeutic approach to manage HPV-positive HNSCC.

## MATERIALS AND METHODS

### TriCurin

Detailed description of the development of TriCurin has been described [42]. Briefly, curcumin, epicatechin gallate, and resveratrol at the molar proportion 4:1:12.5 were prepared at various doses. A solution of 1.28 mM+ TriCurin (1.28 mM curcumin:0.32 mM epicatechin gallate:4 mM resveratrol) in PBS plus 0.1% DMSO was prepared by dilution from solutions of curcumin and resveratrol in DMSO and a solution of epicatechin gallate in PBS. Subsequently, 64 μM+, 32 μM+, 16 μM+, 8 μM+, 4 μM+, 2 μM+ and 1 μM+ TriCurin solutions were prepared through serial dilutions of the 1.28 mM+ in PBS. For the treatment of cells to determine IC50, TriCurin was diluted in the corresponding culture medium.

### Cell culture

UMSCC47 and UPCI:SCC090 HPV16-positive HNSCC cells were obtained from Dr. Thomas Carey (University of Michigan) and Dr. Susanne Gollin (University of Pittsburgh), respectively. Cells were maintained at 37°C, 5% CO_2_ and 95% humidity in Dulbecco's modified Eagle's medium containing 10% (v/v) fetal bovine serum (BioWhittaker, Walkersville, Maryland, USA), 100 units/ml penicillin and 1000 μg/ml streptomycin (Invitrogen, Carlsbad, California, USA). Cell lines were authenticated using short tandem repeat profiling every six months by our research group.

### MTS assay

The sensitivity of cells to TriCurin was measured using the MTT-based colorimetric cell proliferation kit (Roche Applied Science, Mannheim, Germany). Briefly, 3000 cells/well were plated in a 96 well plate. The next day, cells were treated with different concentrations of TriCurin. After 72 hours, 10 μL/well of MTT solution was added to each well and further incubated for 4 hours at 37°C. The formazan crystals formed in the wells were solubilized by adding solubilization solution and incubating the plates for 4 hr at 37°C. The plates were read at 490 nm on a Spectramax 190 plate reader (Molecular Devices Inc., Sunnyvale, CA). The percentage cell growth inhibition for each treatment group was calculated by adjusting the untreated control group to 100%. Data were analyzed using GraphPad Prism software (GraphPad Software, Inc., San Diego, CA) and the dose response curves were used to calculate the concentration of TriCurin resulting in 50% inhibition of cell proliferation (IC50) using a four parametric logistical model. All experiments were repeated at least 3 times.

### Apoptosis analysis

Cells were plated in 6-well plate and incubated with vehicle or TriCurin (3 μM) for 72 hours. Cells were stained with FITC-Annexin V and propidium iodide (Life Technologies, OR, USA) and analyzed by flow cytometry.

### Clonogenic survival assay

Cells were incubated with vehicle or TriCurin for 24 hours (3 μM), harvested and collected, then suspended in complete growth medium. Cells were seeded onto 6-well plates and allowed to grow until visible colonies formed (7–10 days). The cell colonies were fixed with cold methanol, stained with 0.25% crystal violet in 25% methanol, washed and air-dried.

### Tumorsphere formation assay

Cells were incubated with vehicle TriCurin for 24 hours (1, 3, 10 μM), harvested, and collected and washed to remove serum, then suspended in serum free DMEM/F12 supplemented with 1% antibiotic-antimycotic (Life Technologies, OR, USA), 20 ng/mL human recombinant epidermal growth factor (hrEGF), 10 ng/mL human recombinant basic fibroblast growth factor (hrbFGF), 2% B27 supplement (Invitrogen, Carlsbad, CA, USA), 1% N2 supplement (Invitrogen, Carlsbad, CA, USA). Subsequently, cells were cultured in ultralow attachment 96-well plates (Corning Inc., Corning, NY, USA) for tumorsphere formation.

### Quantitative real-time reverse transcription PCR

Real-time reverse transcription PCR was performed using real-time PCR universal reagent and ABI-7900HT real-time PCR machine (Applied Biosystems). All reactions were done in a 20 μl reaction volume in triplicate using validated TaqMan Gene Expression Assays for p53, HPV16E6, HPV16E7, and GAPDH. PCR amplification consisted of an initial denaturation step at 95°C for 10 minutes, followed by 40 cycles of PCR at 95°C for 15 seconds, 60°C for 60 seconds. Standard curves were generated and the relative amount of p53, HPV16E6, and HPV16E7 was normalized to GAPDH, respectively.

### Western blot analysis

Whole cell lysates were mixed with Laemmli loading buffer, boiled, separated by sodium dodecyl sulfate polyacrylamide gel electrophoresis, and transferred to a PVDF membrane. Subsequently, immunoblot analyses were performed using antibodies specific to p53 (Calbiochem, San Diego, CA), HPV16E6 (Abcam, Cambridge, MA), HPV16E7 (Cervimax, Vienna, Austria), or GADPH (Cell Signaling Technology, Danvers, MA). The signal was developed with ECL (Thermo Fisher Scientific) after incubation with appropriate secondary antibodies.

### Pre-clinical model of HPV-positive HNSCC

Athymic nude (NCr) mice were purchased from Charles River Laboratories (Wilmington, MA) and housed at The Ohio State University animal facility. Animals were allowed to acclimate for 1 week prior to experimentation. All animal work performed was in accordance with and approved by the Institutional Animal Care and Use Committee at The Ohio State University. UMSCC47 cells grown to 80% confluence were harvested, and the cells (1 × 10^6^) mixed with Matrigel (1:1) were implanted into the flanks of 8-week old female athymic nude mice and tumors were allowed to develop without treatment. At 41 days after implantation, mice with palpable tumors were assigned to two experimental groups and treated with 0.1% DMSO in PBS-control or 1.28 μM+ TriCurin (5 μL, three times per weekly, *n* = 7) by direct intra-tumoral injection. Tumors were measured using a digital caliper and tumor volumes were calculated using the formula: tumor volume = length × width × height × 0.5.

### Immunohistochemistry

After the treatment protocol, the mice in both experimental groups were sacrificed and the entire tumors were resected. About two-thirds of each tumor was fixed in 4% paraformaldehyde. The formalin-fixed, paraffin-embedded tissues were sectioned at 4 microns and stained with hematoxylin and eosin (H&E) for histologic examination. Immunohistochemistry for Ki67 levels was performed using an antibody against the C-terminal portion of Ki67 (Catalog # 790-2910, Ventana Medical Systems Inc., Tucson, AZ). Immunostaining was performed on an autoimmunostainer (Ventana XT System Benchmark; Ventana Medical Systems) according to the manufacturer's recommendations. Ki67 immunostaining was evaluated by two pathologists. The typical areas for each case were photographed with a 20× objective lens using a Nikon microscope and digital camera DS-L3 (Nikon, Tokyo, Japan). The Ki67 labeling index was scored as the percentage of tumor cell nuclei showing definite nuclear immunoreactivity above the background level using Image J (National Institute of Health). About a third of each excised tumor was processed for immunofluorescence staining for HPV16/18E6. Coronal sections (30 μm) were made from 4% paraformaldehyde-fixed and 30% sucrose-soaked tumors. Tumor sections of three animals from each group were processed as follows for antigen retrieval: incubation with formamide 2 × SSC (1:1), 55°C for 2 h, and then 5 min in 2 × SSC (NaCl and sodium citrate) at room temperature. After blocking overnight at 4°C in 0.1 % Triton X-100, 3% goat serum in 100 mM phosphate buffered saline (PBS), the sections were treated overnight with primary antibody: HPV16/18 E6 (C1P5; sc-460) (1:100), (diluted in 2% goat serum and 0.1% Triton X-100 in PBS). Secondary antibody control sections were incubated overnight at 4°C in blocking solution. After washing three times with PBS, the goat anti-mouse-488 secondary antibody (Invitrogen-Molecular Probes) (1:1000 dilution) was added to the primary antibody-treated as well as secondary control sections. Following overnight incubation at 4°C and three washes with PBS, the sections were treated with HOECHST33342 (10 μg/ml) for 30 min at room temperature. Next, the sections were washed three times with PBS and mounted on microscope slides with Gold anti-fade mounting fluid (Invitrogen-Molecular Probes). Confocal imaging was conducted using a Leica SP2 microscope. ImageJ was used to obtain the HPV16/18E6 and HOECHST33342 fluorescence intensity. HPV16/18E6 intensity (green) was normalized to HOECHST33342 intensity (blue).

### Statistical analysis

The statistical significance of the results was evaluated using ANOVA and a Student's *t*-test. *P*-value < 0.05 was considered significant.

## SUPPLEMENTARY MATERIALS FIGURES


