# Supplementary material for: TriCurin, a novel formulation of curcumin, epicatechin gallate, and resveratrol, inhibits the tumorigenicity of human papillomavirus-positive head and neck squamous cell carcinoma
Source: Oncotarget. 2016 Jul 16;8(36):60025–35. doi: 10.18632/oncotarget.10620 (PMC5601119; doi:10.18632/oncotarget.10620)
Supplement: Supplementary file 1 [file oncotarget-08-60025-s001.pdf]

# TriCurin, a novel formulation of curcumin, epicatechin gallate, and resveratrol, inhibits the tumorigenicity of human papillomavirus-positive head and neck squamous cell carcinoma

## Supplementary Materials

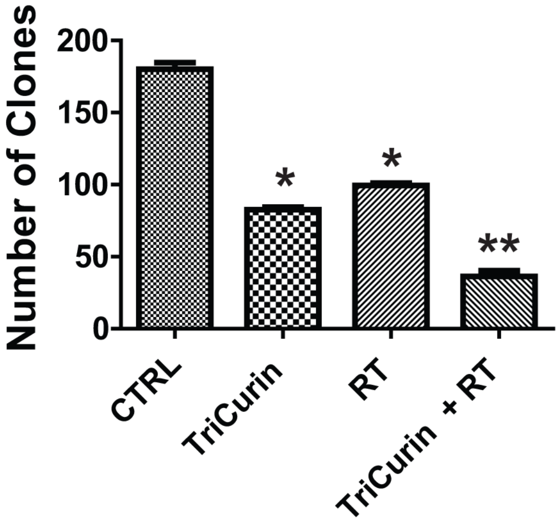


**Supplementary Figure S1: TriCurin potentiates the anti-tumor efficacy of radiation in HPV-positive HNSCC.** UMSCC47 cells were treated with control, TriCurin (3 μM+), radiation (3 Gy), or TriCurin (3 μM+) and radiation (3 Gy). The number of surviving clones was assessed by counting the number of clones under a microscope after crystal violet staining. Data are presented as mean ± SEM. **P* < 0.01, *n* = 3; TriCurin or radiation vs. control; ***P* < 0.01; TriCurin + radiation vs. TriCurin or radiation.
